# The effect of karate interventions on the motor proficiency of female adolescents with developmental coordination disorder (DCD) from high and low socio-economic status

**DOI:** 10.1186/s13102-022-00501-6

**Published:** 2022-07-06

**Authors:** Farhad Ghadiri, Wesley O’Brien, Sana Soltani, Marzieh Faraji, Moslem Bahmani

**Affiliations:** 1 Faculty of Physical Education and Sport Sciences, Kharazmi University, Mirdamad Blvd., Hesari St, Tehran, Iran; 2 School of Education, Sports Studies and Physical Education Programme, University College Cork, 2 Lucan Place, Western Road, Cork, Ireland

**Keywords:** Girls, Motor skills, Movement impairment, Social class, Martial arts

## Abstract

**Background:**

There is not a general consensus on the best type of intervention to improve the motor proficiency (MP) of adolescents with developmental coordination disorder (DCD). Considering the effect of socio-economic status (SES) in relation to the MP of adolescents with DCD, it is necessary to examine this issue further. The purpose of this study was to investigate the effect of Karate-Do interventions on the MP of adolescents with DCD from high/low SES.

**Methods:**

Participants included 16 adolescent girls (12 to 13 years old) with DCD, and their classification into high/low SES groups was done by using appropriate previously validated questionnaire. A short form of the Bruininks-Oseretsky Test of Motor Proficiency, Second Edition (BOT-2), was used to assess fine motor precision, fine motor integration, manual dexterity, upper limb coordination, bilateral coordination, balance, strength, speed, agility, and overall MP level of the participants. Both high/low-SES groups completed a specifically prescribed Karate-Do intervention program for 8 consecutive weeks (3 sessions per week with each session lasting for 75 min).

**Results:**

Results from this 8-week intervention showed that the pattern of change in manual dexterity, bilateral coordination, strength and the total MP score improved over time in both groups with varied socioeconomic backgrounds. Specifically, the high-SES group performed significantly better than their low-SES counterparts during the 8-week Karate-Do intervention (*p* < 0.05).

**Conclusions:**

Following participants’ completion of the Karate-Do intervention, the existence of significant changes in the MP of adolescents’ with DCD at high/low SES confirmed our hypothesis. Compared to the low-SES group, adolescents with high SES displayed superior MP following the intervention. It seems that karate (kata) training serves as a good alternative for rehabilitation MP programs, however, an important issue concerning social infrastructure is to create a suitable exercise environment for adolescents at lower SES. Until the SES achievement gap in female adolescent MP is stable, future work is warranted to discover more practical and meaningful interventions.

**Supplementary Information:**

The online version contains supplementary material available at 10.1186/s13102-022-00501-6.

## Background

Developmental coordination disorder (DCD) is considered as a specific developmental disorder of motor function, as characterized by impairment in motor coordination and clumsiness in both fine (handwriting and shoelace tying) and gross (playing sport and getting dressed) motor skills [1]. This disorder, which affects 5-6% of school-aged children [2, 3], interferes with youth’s higher-order cognitive functions such as executive function and academic performance [4, 5]. Furthermore, DCD can affect various aspects related to individuals’ physical health and psycho-social well-being (e.g., cardiovascular fitness, muscle strength, physical activity participation, self-efficiency, perceived competence, self-worth, social connections, etc.) [6–8]. Some of these effects seem to be pervasive and enduring during subsequent years [3]. For example, it has been suggested that in more than half of children with DCD, problems persist into adolescence and adulthood [2, 3]. However, intervention studies have mainly focused on typically developing children so far, and little attention has been paid to the effects of motor interventions on motor proficiency (MP) of adolescents with DCD [9].

According to the International Classification of Function, Disability, and Health for Children and Youth (ICF-CY) framework [10], motor interventions are categorized into three types based on the aims that an intervention follows: (1) *body function & structure-oriented interventions*, where an activity or intervention is designed to improve body functions and considered to underlie the identified functional problems (e.g., strength training, biofeedback training, visual training, balance-board training, etc.); (2) *activity-oriented interventions,* where the activity is designed to improve performance in that activity (e.g., sport/play-related training, virtual reality training, active video game training), and (3) *Participation-oriented interventions*, where an activity or intervention is designed to increase participation in that activity (see also Smits-Engelsman et al. [11]). Regardless of the aim that an intervention follows, there is a clear gap in the scientific knowledge regarding how adolescents with DCD respond to motor interventions. As an exception, Bonney et al. [12] compared the effects of two different types of interventions (task-oriented functional training vs Wii training) on MP and activities of daily life in adolescents with DCD. They found that both types of interventions were effective in improving several outcome measures (e.g., muscular strength, running and agility performance, total MP, the tendency for physical activity participation, etc.).

Difficulties in coordinating motor actions in individuals with DCD have been mainly attributed to impaired postural control, sensory-motor integration, muscular strength, speed, and agility [13–15]. Therefore, it is important to apply interventions that target these factors. In this regard, studies suggest that regular practice of martial arts (e.g., Karate-Do, judo, taekwondo) improves children's postural control and balance abilities as an important component of MP [16, 17]. A recent review showed that regardless of the individual's age of commencement of practice, martial arts can improve balance, muscular strength, and cognitive function of healthy adults [18]. It has also been found that karate and judo athletes have faster reaction times than non-athletes [19]. These benefits were also observed in children with DCD, and an improvement in sensory organization and standing balance has been observed after a taekwondo intervention [20]. Moreover, studies suggest that an increase in isokinetic knee muscle strength at 180º and static single‐leg standing balance in DCD children can be obtained after 3 months of intensive taekwondo practice [21]. Considering that karate requires a high level of coordination for executing precise techniques under static and dynamic conditions, in this study, the research team were interested in examining the effects of Karate-Do on MP in adolescents with DCD [22], because karate practice also requires a high level of motor and functional abilities involving speed, strength [23], and precise control over executed movements [24–26]. A recent study has revealed that prolonged karate training can result in superior functional balance in adolescents, suggesting that moderate karate/kata training would be a good alternative in rehabilitation programs [27]. Although several studies have been conducted to uncover the effects of Karate-Do interventions on motor performance, most of the previous studies have focused only on the effects of interventions on balance performance, and few studies addressed the effects of Karate-Do interventions on motor proficiency, especially in adolescents with DCD.

In addition, the ecological perspective, in contrast to the maturational perspective, assumes that all changes in human motor behavior are not attributable to human body systems (e.g., central nervous system), with other factors such as the environment significantly contributing to manifesting behavior. Thus, both the body and the environment are important when a researcher attempts to understand the effects of a particular intervention on motor outcomes. The socio-economic status (SES) of a family is an environmental factor that is known to influence MP [21–26]. Hardy et al. [28] in a study using 6917 students (52% for high school students) found that girls with low SES were twice as likely to be less competent in locomotor skills, when compared with high SES peers [27]. Klein et al. [29] also found that children and adolescents with a higher SES exhibit a higher MP (e.g., in jumping and running tests) than their peers with a lower SES background [30]. It has also been demonstrated that high SES children exhibit superior fine and gross MP compared to middle- and low-SES children [21]. However, a recent study has reported that the effect of SES on motor scores (e.g., fine motor, gross motor, and balance performance) is neglectable [31]. In the existing literature, few studies have tried to find out if SES impacts the MP levels of adolescents with DCD. Valentini et al. [32] found that 9–10 year-old children with probable DCD and at risk of DCD exhibit an inferior performance in balance and manual dexterity tasks. More importantly, in that study, SES was found to be the strongest predictor of motor outcomes, and low SES was associated with significantly poorer MP [33]. Considering the discrepancy in the MP levels of children with different SES, it is reasonable to expect that SES modulates the effects of motor interventions.

In sum, the current knowledge regarding the effects of motor interventions on motor performance in adolescents with DCD is highly limited. In addition, while several studies suggest that SES impacts children’s MP, few studies have attempted to investigate the effect of environmental factors, including SES, on the performance of adolescents with DCD. Therefore, in this study, the research team aimed to investigate the effects of an 8-week karate intervention on the MP in adolescents with DCD and high/low SES. As studies suggest that martial arts, including karate, may improve factors such as balance performance [30], muscular strength, endurance, speed, and agility, the research team hypothesized that these factors would improve following our karate intervention, and that the rate of improvement would vary depending on the level of participants' SES. The research team further hypothesized that the karate intervention would result in adolescents with DCD exhibiting an improved performance in measures, such as fine motor precision and manual dexterity. The later hypothesis is based on several studies which suggest that cognitive factors, such as executive function, selective attention, and reaction time improve following karate practice [18, 31].

## Methods

### Participants

Participants were recruited from two high schools in different neighborhoods of Tehran (Iran). In Tehran, neighborhoods are classified into five quintiles based on different socio-economic indicators [33]. In the present study, two schools were randomly selected from both ends of the quintile where Quintile 5 represented the “totally developed neighborhoods” and Quintile 1 was classified as the “in the need of intervention neighborhoods”. For ease of exposition, quintiles are referred to hereafter as ‘‘high-SES’’ (totally developed) and ‘‘low-SES’’ (in need of intervention). In addition to the community-based measure for determining SES level, the family background of adolescents was also measured using the Persian version of a SES scale [34] to make sure that the family background suitably matches the type of school environment (high-SES or low-SES).

From the list of students in high- and low-SES groups, those identified by general practitioners, teachers, school health nurses, a consultant pediatrician, and a health visitor (nurse specialist with qualifications in community health) were assessed based on the diagnostic criteria for DCD according to the Diagnostic and Statistical Manual of Mental Disorders, fifth edition (DSM-5) [2, 35]: (1) lower than the 5th percentile as evaluated with the Movement Assessment Battery for Children-2nd Edition (M-ABC2; [36]) (criterion A); (2) lower than the cut-off point as evaluated by the revised version of the Developmental Coordination Disorder Questioner (DCDQ-7; [37]) (criterion B); (3) onset of symptoms early in development (criterion C); and (4) no diagnosis of a general medical condition (e.g., cerebral palsy, hemiplegia, and muscular dystrophy), visual impairment, or intellectual disability (criterion D). Medical qualifications were confirmed by interviewing the parents or guardians of participants, as well as further regular medical checkups by a pediatrician. The M-ABC2 was administered by a specialist in the field of children's motor development who was fully trained on testing motor development. These assessments were performed after parents expressed their informed consent.

Following the rollout of this assessment protocol for DCD, eight female adolescents from a low-SES background (12.37 ± 0.51 years) met our inclusion criteria and therefore were included in the study. Regarding high SES, nine adolescents met the inclusion criteria. One adolescent was unable to attend the intervention sessions, leaving a final sample of eight female adolescents (12.50 ± 0.53 years) from a high-SES background also included in the study.

The experimental procedures were approved by the Institutional Review Board of Kharazmi University. There were no foreseeable risks to the participating adolescents. All personal identification information on SES and DCD was entirely confidential.

Prior to data collection, the research team explained the purpose of the study to the adolescents and their parents/guardians. Given the age range of participants, parental/guardian written informed consent was obtained prior to participation in this study. The procedures complied with ethical standards of the Declaration of Kharazmi university on the treatment of human subjects in research (approval number: IR.UT.PSYEDU.REC.1399.013).

### Measures

#### Demographic information and physical activity

Basic demographic information was acquired through individual interviews with each of the participants. These interviews were conducted by the fourth author based on previously designed and validated questions [38]. Such questions were related to age, general health, disease record, disorder, previous sports background, and time of menarche. The International Physical Activity Questionnaire (IPAQ-A) was used to measure the level of physical activity [39]. This questionnaire, which was completed by each participant, uses the information obtained to report the level of energy expenditure of each individual in metabolic equivalents (METs) minutes per day.

#### SES questionnaire

When measuring the socio-economic background of a family, the Persian version of a SES questionnaire was used [34], which consisted of four components relating to income, economic class, education, and housing status, along with a total of six demographic questions and five general questions. After a full explanation of the questionnaire and the data confidentiality protocol of the questionnaire, each participant’s parent/guardian filled out a paper questionnaire in the school, and Parents and guardians were asked to fill out the questionnaire in 15 min using a Likert Scale for investment income, education, social class, and housing prices. For consistency of community-based criteria for SES and family background, participants were considered low SES if the total score of the parents' answers to the questionnaire was between 5 to 8 and high SES if the total score was between 22 to 25. The validity and reliability of the questionnaire were confirmed by 12 experts, supported by a Cronbach's alpha of 0.83 [40].

#### BOTMP-SF2 test

The short version of the Bruininks-Oseretsky Test of Motor Proficiency Second Edition (BOTMP-SF2) [41] was completed for evaluation of the MP of adolescents in pre- and post-testing phases by the fourth author, who had expertise in motor development. This validated measurement tool assesses fine motor precision, fine motor integration, manual dexterity, upper limb coordination, bilateral coordination, balance, strength, speed, and agility and provides a single overall motor composite score. To meet the requirements of the testing protocol, the test was administered in a calm atmosphere, in the school gym with dimensions of 10 m × 30 m. The time for taking the BOTMP-SF2 was approximately 15 min per participant. The BOTMP-SF2 was previously validated against the long version of the original battery for people aged 4 to 21, and the usefulness of this tool was confirmed for the motor assessment, development, and evaluation of motor intervention programs [41]. The BOTMP-SF2 subtest scores are age-adjusted scaled scores, possessing a mean of 15 and a standard deviation of 5, whereas the BOT-2 composite scores are standard scores (derived from summing the subtest scale scores and converting them into a quotient) with a mean of 100 and a standard deviation of 15. Scaled scores were converted into standard scores. The reliability of the BOTMP-SF2 was reported to be between 0.89 and 0.90 [42].

### Procedure

The intervention program used in this study was adopted from the published Karate-Do guide [43]. The Karate-Do intervention was conducted by an expert Karate-Do instructor who was an official coach of the Karate Federation. The Karate-Do intervention involves performing the Kihon and Heian Shodan kata. Each session of intervention program targeted the development of balance, coordination, and strength for a total of 75 min, including a 45-min episode of the Karate-Do Martial activity. Each session included 30 min for the warm-up and cool-down exercises (see Table 1). In the early sessions of the intervention, the exercises and activities were taught in a simple and direct pedagogical manner. The exercises were performed in smaller groups with the instructor in order to enhance the fluidity of the sessions. At the initial stages of learning new movements, all participants were required to slow down and control their movements. Advancing the practice of resident gestures was static to dynamic movements. Also, the periods of rest between each workout to the next exercise were considered. The intervention lasted for 8 weeks.

**Table 1:** Eight-week program followed by the groups

Duration (time) [min]	Section	Activities performed during each training session
5	Introduction	Seiretsu (Line up), Kiotsuke (stand at attention), Rei (bow) and Yoi (pay respects to the instructor). Sit in meditation (breathing exercises), Focus Claps.
15	Warm-up	Exercises including running, hopping, jumping, etc. Stretching activities: hamstrings, hip flexors, lower back, calves, shoulders, and chest stretches.
20	Basic technical skills (i.e., Kihon)	Tachi Kata (Stances): shizentai (natural stance), zenkutsu dachi (front stance), kiba dachi (straddle stance), kokutsu dachi (back stance) Uke (Blocks): gedan barai (downward block), lower sweeping (block jodan), age uke (face block), chudan soto uke (outside middle block), chudan uchi uke (inside middle block), shuto uke (knife hand block). Tsuki (Punches): oi zuki (lunge punch), gyaku zuki (reverse punch) keri (Kicks): mae geri (front kick), mawashi geri (roundhouse kick)
25	Pre-arranged sequence of techniques and movement against visualized opponents (i.e., Kata)	Heian Shodan Kata. Progressive complex sequence development over the eight weeks of the intervention.
10	Cool down	Light jogging or walking, Upper body stretch, Knee-to-Chest Pose, Reclining Butterfly Pose, Child’s Pose.

### Statistical analysis

The results of fine motor precision, fine motor integration, manual dexterity, upper limb coordination, bilateral coordination, balance, speed, agility, and strength tests were recorded on two occasions during the pre- and post-assessment stages. Scaled scores from the individual 8 items of the BOTMP-SF were obtained, and the total scores were calculated for further analysis.

Repeated measures analyses of variance (ANOVA) were conducted to examine the effect of the Karate-Do intervention on the MP of high- and low-SES groups. The η^2^_p_ values were reported for the effect size and considered small for η^2^_p_ < .06, moderate for η^2^_p_ > .06 and η^2^_p_ < .14, and large for η^2^_p_ > .14 [44]. The typical assumptions of ANOVA, including data normality as well as sphericity and homogeneity of variance, were checked by boxplot and Q–Q plot (residuals vs fitted values). All participants completed the intervention, and the data obtained at pre- and post- assessments were submitted to statistical analysis. The analyses were conducted using SPSS, version 23.0, with the significance level being set at *p* ≤ 0. 05.

## Result

The information on the participants’ demographic characteristics and the values for body mass index are provided in Table 2.

**Table 2:** Demographic characteristics and body mass index values (mean and standard deviations) of high and low SES groups

Variable	Low SES	High SES	T	*p* value
Age (year)	12.37 (0.51)	12.5 (0.53)	0.67	0.51
Height (cm)	157.32 (10.09)	158.16 (6.64)	0.20	0.84
Weight (kg)	54.8 (0.47)	55.2 (0.47)	1.59	0.13
Body Mass Index	21.56 (1.40)	22.37 (3.4)	0.618	0.54
Total PA (MET min day^−1^)	612.2 (68.23)	614.6 (71/09)	0.68	0.94
Maturity	Post menarche	Post menarche		
Movement Assessment Battery for Children—Second Edition (percentile)	3.56 (0.49)	3.86 (0.63)	1.04	0.312

*SES* socio-economic status, *cm* centimeter, *kg* kilogram.

There were no differences in the baseline MP performances between high- and low- Regroups (see Table 2).

## Effect of intervention on motor proficiency

**Fig. 1:**
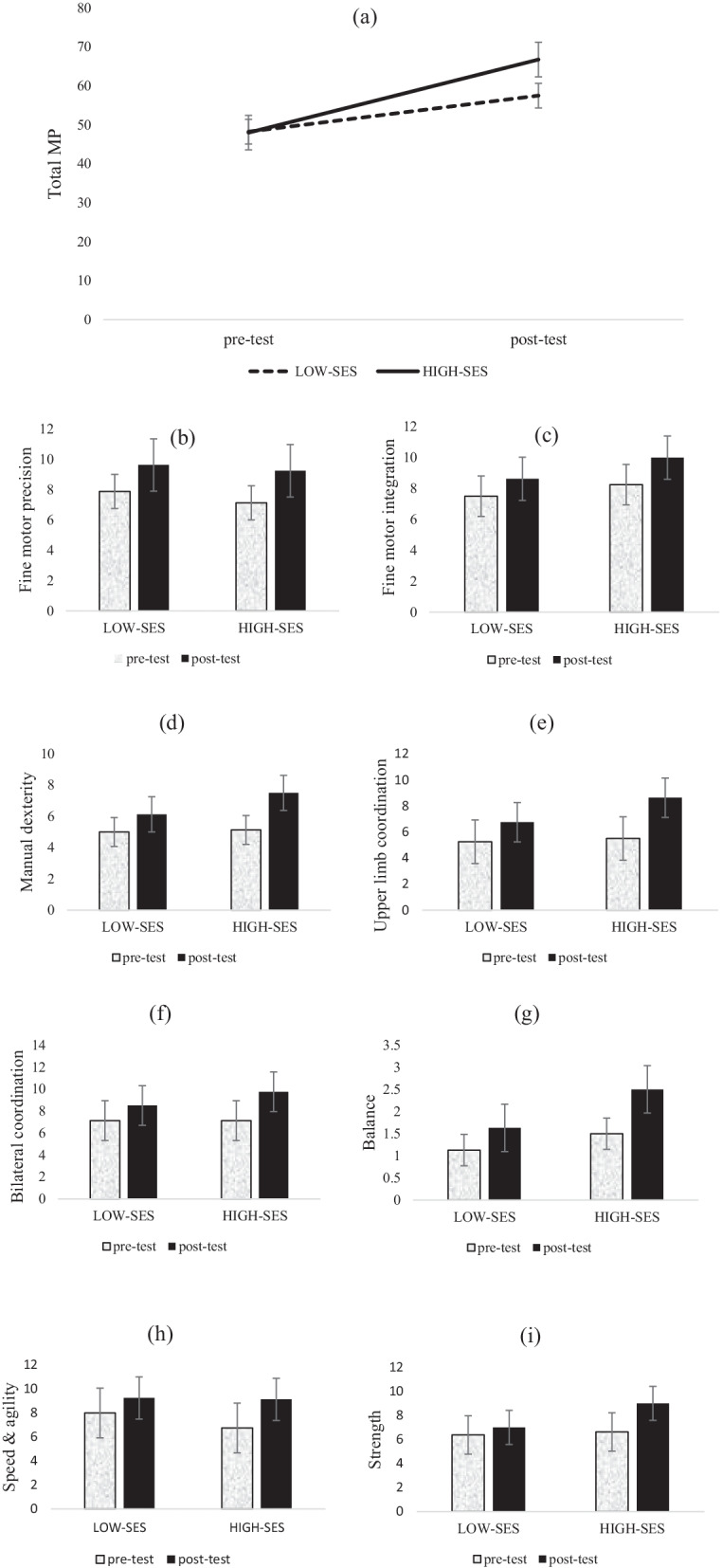
**a** Total MP score, **b**–**i** subscales scores

The results of a 2 × 2 repeated measures ANOVA, as presented in Table 3, revealed that there were significant main effects for time across all eight subscale scores and the total MP score (*p* ≤ 0.01). The interaction between group and time was significant for fine motor integration, manual dexterity, bilateral coordination, balance, strength, and the total MP score (*p* < 0.05). Post-hoc analyses using Bonferroni adjustments revealed that while there were no differences between low and high SES scores at the baseline, the high SES group improved more significantly in fine motor integration, manual dexterity, bilateral coordination, balance, strength, and the total MP than low our SES group (*p*s < 0.05; see Fig. 1a–i) for more details see the Additional file 1.

**Table 3:** Pre- and post-test MPcomparisons between high and low SES groups

Subscales	Source	F	*P*	Partial *η*2
Fine motor precision	Time	131.902	.000	.904
	Time * group	1.235	.285	.081
Fine motor integration	Time	194.895	.000	.933
	Time * group	9.211	.009	.397
Manual dexterity	Time	53.804	.000	.794
	Time * group	6.863	.020	.329
Upper limb coordination	Time	42.353	.000	.752
	Time * group	4.079	.063	.226
Bilateral coordination	Time	174.236	.000	.926
	Time * group	21.509	.000	.606
Balance	Time	115.613	.000	.892
	Time * group	11.290	.005	.446
Speed and agility	Time	21.000	.000	.600
	Time * group	2.333	.149	.143
Strength	Time	65.032	.000	.823
	Time * group	22.129	.000	.612
Total Score	Time	1774.957	.000	.992
	Time * group	175.913	.000	.926

## Discussion

The purpose of this study was to investigate the effect of a Karate-Do intervention program on the MP of adolescent girls with DCD at high/low SES. To this end, we investigated the effect of Karate-Do training for 16 sessions in 8 weeks. Confirming one of the study hypotheses, Karate-Do intervention was found to significantly improve the MP of both high- and low-SES groups over time. This finding is consistent with a line of research, which suggests that martial arts can improve the MP of children and adolescent youth with DCD [20, 21, 45]. Moreover, our findings extend the literature base, as to the authors' knowledge, this is the first study reporting that Karate-Do exercises can benefit the MP of female adolescents with DCD.

Studies found that active video game (AVG) interventions are effective in improving balance [46], and General Skill Training may improve strength or endurance [47]. These studies along with our findings suggest that activity-oriented interventions (e.g., sport/play-related interventions) are generally effective in improving body functions [11]. Activity-oriented interventions, including sport-related interventions, are generally more cost-effective than interventions directed to body functions and structures, because the latter usually needs relatively expensive training devices (e.g., biofeedback, eye-tracking systems, resistance-training devices, etc.). A good avenue for research in DCD is to investigate whether activity-oriented interventions can improve body functions and structures at a comparable or even a superior level to interventions targeting body functions and structures. Interestingly, the current study observed that following the Karate-Do intervention, body functions (such as balance and muscular strength, as well as manual dexterity, fine motor precision, and fine motor integration) improved. While improvements in balance, muscular strength, and bilateral coordination are to be expected after a karate intervention, it may be challenging to explain why factors such as fine motor integration and manual dexterity have improved in this study. There is a need for providing further direct evidence, however, these findings may be explained from cognitive and neural perspectives. It has been shown that cognitive functions such as executive functions, attention, and reaction time improve following Karate-Do practices. Furthermore, some studies suggest that karate improves motor control due to the microstructure of white matter in the cerebellum and M1 [48]. Further studies, however, are needed to arrive at robust conclusions as to how cognitive and neural factors contribute to the MP improvements in adolescents with DCD who have engaged in karate practice.

As mentioned earlier, female adolescents with DCD at high-SES group had an increased likelihood of demonstrating significantly better improvements in overall MP over time when compared to the low-SES group. Along similar lines, a research by Tine and Butler [49] reported that children at low SES are susceptible to additional benefits in acute-based aerobic exercises involving selective attention. Golos et al. [50] also found increased intervention effects in both cognitive and motor skills of boys at low SES. The inconsistency in these findings may be attributable to the difference in the age range of participants in our study and the above studies. While SES in adolescence appears to have a significant moderating role in the effectiveness of motor interventions, its function may be different between children and adolescents.

During the period of maturation, adolescents of lower SES background appear to consistently experience more stressful and less cognitively stimulating situations at the home environment, when compared to their high-SES peers [51–54]. In addition, studies show that SES is positively associated with motor competence [55]. For example, it has been shown that children and adolescents with low SES are less competent in fine and gross motor skills, as well as stability performance, when compared with high SES peers [28, 29, 56]. It is evident from the present study findings that SES has a significant role in adolescents’ response to activity-oriented interventions.

The analyses of the subcomponent scores revealed that the two SES groups significantly differed in manual dexterity, bilateral coordination, and strength. Manual dexterity is one of the main components of fine-motor skills, and in previous studies, it has been found that social disadvantage may have a persistent, detrimental effect on this skill over time [57]. Regarding the motor coordination, Prätorius and Milani. [58]. have further shown that children with lower social backgrounds are at an increased likelihood of coordination impairments, and these abilities have not changed significantly within the last 25 years. Such pieces of evidence can help explain the current research findings in terms of low-SES participants' MP improvement after 8 weeks of e Karate-Do intervention.

This study has several limitations that lead to recommendations for future studies. Due to the limited number of adolescent participants with DCD at high/low SES, a generalization of the results to other young people should be made with caution. In addition, while the researcher who collected participants’ data was an experienced motor development evaluator, she was not blind to the groups’ division (low vs high SES), which may have led to some unintentional effects (i.e., biases) on our collected data. Future studies are required to consider such potential limitations. The study is only limited to adolescent girls with DCD, and as a result, the research team failed to include adolescent boy counterparts. As it has been shown that there are gender differences in MP in individuals with DCD, future studies using both genders are required. In order to determine the effects of SES as an environmental factor, it is recommended to study the long-term effect of motor development interventions across different age groups with other developmental disorders.

## Conclusion

Due to the limited research in the field of cognitive and motor functioning in adolescents with DCD and high/low SES, it is expected that the current findings can be used to facilitate low-cost interventions that can be implemented in schools. Such findings have the potential to provide motor development opportunities for adolescents with DCD, especially those possessing high SES.

## Supplementary Information

**Additional file 1.** Pre- and post-test data for Motor Proficiency (Bruininks-Oseretsky Test) between High and Low Socio-Economic Status groups after Karate Interventions.

## Data Availability

All data generated or analysed during this study are included in its Additional file 1.

## Abbreviations

MP: Motor Proficiency
DCD: Developmental Coordination Disorder
SES: Socio-economic status
DSM-5: Diagnostic and Statistical Manual of Mental Disorders, fifth edition
M-ABC2: Movement Assessment Battery for Children-2nd Edition
DCDQ-7: Developmental Coordination Disorder Questioner
IPAQ-A: International Physical Activity Questionnaire
METs: Metabolic equivalents
BOTMP-SF2: Bruininks-Oseretsky Test of Motor Proficiency Second Edition
ICF-CY: International Classification of Function, Disability, and Health for Children and Youth
AVG: Active Video Game
